# Case of Prolapsus Iridis

**Published:** 1851-02

**Authors:** G. R. Morehouse

**Affiliations:** Philadelphia


*Case of Prolapsus Iridis.* By G. R. Morehouse, M. D., Philadelphia.

James Mahoney, æt. 19, on the morning of Dec. 4th, while engaged at his occupation of sail making, received a severe injury of the cornea in the following manner. In attempting to pass his needle through a tense seam of sail-cloth, it slipped from his grasp and was thrown by the recoil of the seam against the cornea of the right eye, penetrating it midway between its

*Muller's Phys. Vol. I. pp. 550. Beaumont, p. 113. Carpenter's Principles of Physiology, p. 490. Magendie, p. 290.

†Blondlot, Traité Analytique de la digestion.

centre and external border. The needle was almost immediately withdrawn from it position, by its own weight, aided by the movements of the patient. About two hours after the occurrence of the accident, I saw the case for the first time ; the patient then complained of moderate pain, intolerance of light and excessive lachrymation. Upon examination it was found the needle had cut its way through the membrane of Descemet, as well as through the cornea, making an incision of about one-eighth of an inch in length, very nearly in the line of the transverse axis of the eye. A great portion of the aqueous humor had been evacuated and still continued to ooze from the opening when the head was placed in a favorable position. A fold of iris was protruding, forming a flat triangular projection, the base of the triangle coinciding with the line of incision, and its inner boundary being formed by the pupillary margin of the iris ; it was between the two layers of this free border that the aqueous humor continued to escape. The pupil was much elongated, its external margin terminating in the point where the fold of the iris protruded through the cornea. The color of the projecting portion was not sensibly different from that natural to the iris of the right eye. The iris of the left eye could not be compared, having been darkened by previous disease. As might have been anticipated, from its color remaining unchanged, the protrusion was found, upon examination with a pocket glass, but slightly congested, probably owing to the line of incision corresponding in direction with the course of its principal vessels.

Under the circumstances which this case presented, the indication was evidently to restore the iris *whole* to its natural position, if possible, and if not, to snip off the protruding portion, preferring an enlarged and irregular pupil, rather than opacity of the cornea with synechia anterior, which would have inevitably resulted, had the iris and cornea been permitted to remain in apposition. To aid, therefore, in the reduction, as well as to prevent further effusion of the aqeuous humor, the patient was put upon his back, and with the hope of producing dilatation of the pupil and consequent retraction of the protruded iris, a solution of the active principle of belladonna in the following proportions was applied, by means of a camel's-hair pencil to the conjunctiva of the injured eye:

R. Atropiæ Sulphat, gr. v.

Aquæ distillatæ, f.℥j.

The iris, however, being deprived of the support of the aqueous humor, did not respond so markedly as is usual, to the action of the atropia. It was therefore thought necessary to repeat the application, and in this instance it was made to both eyes with much increased affect. Cold water was also permitted to drop on the iris—to reduce the size of the protrusion as much as possible. Under this treatment, the iris, in half an hour from the first application, had entirely withdrawn itself within the orifice, and resumed its natural position, the lips of the wound in the cornea were pressed together and their edges touched with nitrate of silver. The patient was ordered to remain upon his back—but lest he should fail in doing so, the extract of belladonna reduced to the consistence of honey was smeared over the eyelids and about the orbit, to keep the pupil continually dilated.

On the morning of the next day, the wound was doing well; the photophobia was very slight; the patient, however, complained of pain in the temples and about the orbit; the change in color of the reduced portion of the iris was more marked than at first. An active cathartic was prescribed to counteract the tendency to inflammation. In the evening—the pain was less severe, the bowels had been copiously moved. On the following day all the symptoms were much improved, in the iris the incision the cornea had healed, the difference of color was much less marked, and the pain had entirely gone. The patient recovered in a short time, the functions of the iris remaining perfect, and the cornea free from opacity.

